# Microbial Genomics as a Catalyst for Targeted Antivirulence Therapeutics

**DOI:** 10.3389/fmed.2021.641260

**Published:** 2021-04-13

**Authors:** Vitali Sintchenko, Verlaine Timms, Eby Sim, Rebecca Rockett, Nathan Bachmann, Matthew O'Sullivan, Ben Marais

**Affiliations:** ^1^Marie Bashir Institute for Infectious Diseases and Biosecurity, The University of Sydney, Sydney, NSW, Australia; ^2^Centre for Infectious Diseases and Microbiology—Public Health, Westmead Hospital, Westmead, NSW, Australia; ^3^Centre for Infectious Diseases and Microbiology Laboratory Services, NSW Health Pathology—Institute of Clinical Pathology and Medical Research, Westmead, NSW, Australia; ^4^Children's Hospital at Westmead, Westmead, NSW, Australia

**Keywords:** virulence, genome sequence analysis, antibacterial treatment, surveillance, antimicrobial stewardship

## Abstract

Virulence arresting drugs (VAD) are an expanding class of antimicrobial treatment that act to “disarm” rather than kill bacteria. Despite an increasing number of VAD being registered for clinical use, uptake is hampered by the lack of methods that can identify patients who are most likely to benefit from these new agents. The application of pathogen genomics can facilitate the rational utilization of advanced therapeutics for infectious diseases. The development of genomic assessment of VAD targets is essential to support the early stages of VAD diffusion into infectious disease management. Genomic identification and characterization of VAD targets in clinical isolates can augment antimicrobial stewardship and pharmacovigilance. Personalized genomics guided use of VAD will provide crucial policy guidance to regulating agencies, assist hospitals to optimize the use of these expensive medicines and create market opportunities for biotech companies and diagnostic laboratories.

## Introduction

Many first-in-class therapies are now entering the global marketplace, evolving at a rapid pace and fueled by advances in genetics and informatics and expectations of precision medicine (1, 2). Some “N-of-1” personalized therapies can be rationally designed and tailored to a particular patient (3). Between 2015 and 2019, a quarter of US FDA approvals for first-in-class' antimicrobial agents have been so-called virulence arresting drugs (VAD). VAD are an expanding class of therapies that act to “disarm” rather than kill pathogens (4, 5), and because most virulence traits are non-essential for bacterial survival VAD are less susceptible to the development of antimicrobial resistance (6). They can restore or augment the effect of traditional antibiotics, but are more pathogen-specific and generally spare the body's healthy bacteria (4).

**Figure 1:**
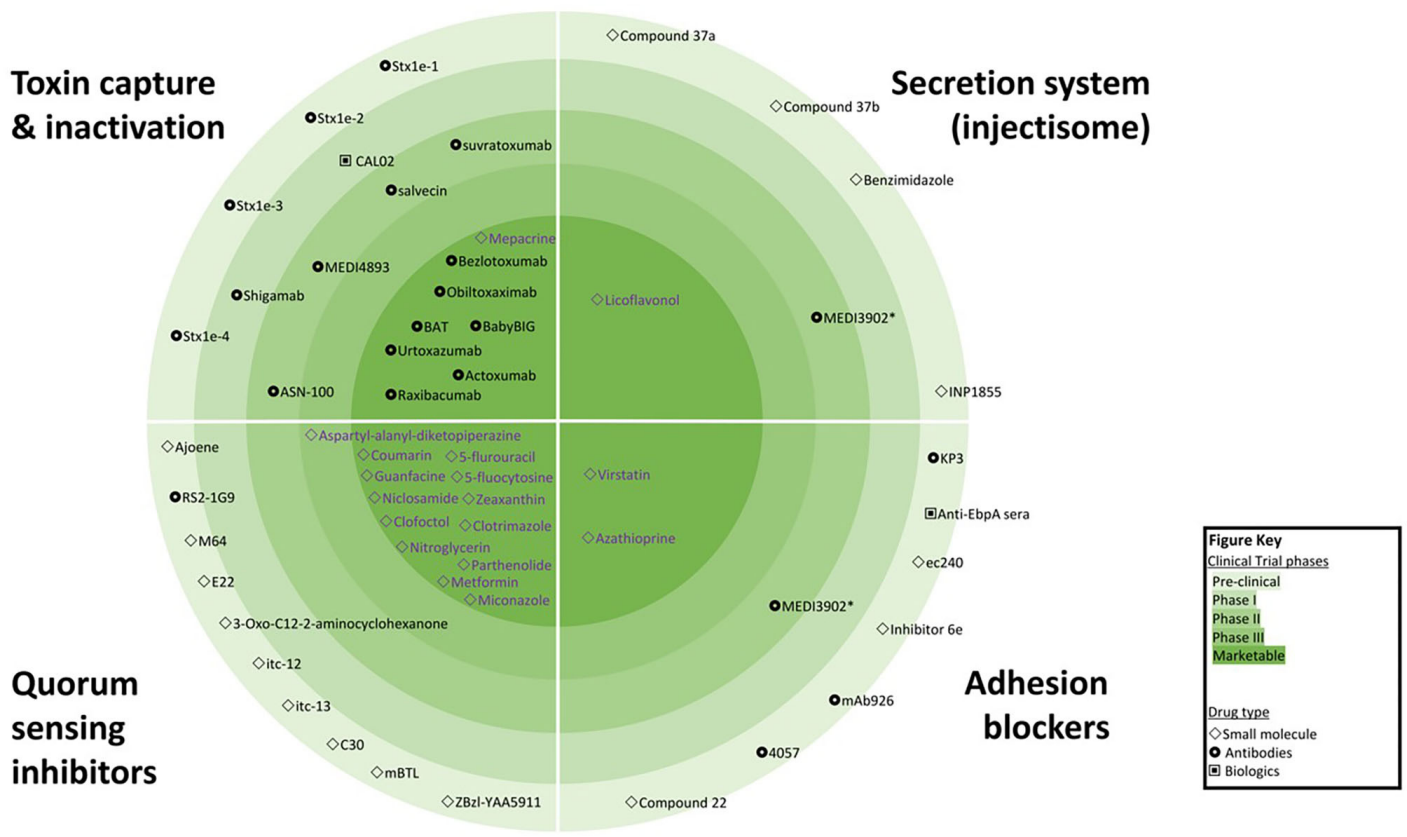
Overview of the competitive landscape for virulence arresting drugs (VAD).

Treatments focusing on anti-virulence effects are not a new idea—the first Nobel Prize in Medicine was awarded in 1901 for the discovery of diphtheria antitoxin. However, in recent years, recombinant DNA technology has revolutionized the scope of anti-virulence strategies, with multiple new drugs showing efficacy and safety in clinical trials (5, 7) and the possibility of engineered antimicrobials offering the prospect of selective killing of target bacteria (8). In total, more than 100 VAD candidates had been reported; seven are already registered by the US Federal Drug Agency (FDA) and/or European Medicines Agency (EMA), while eight more are in Phase II/III clinical trials. In addition, 40 re-purposed drugs with established safety profiles and registered with the FDA/EMA for the treatment of non-infective conditions may hold VAD promise. Figure 1 captures the most advanced classes of VAD with examples of agents available for clinical use or in clinical trials. However, there are also other virulence mechanisms targeted by VAD candidates such as bacterial metabolism (9, 10) and virulence transcription regulation (11, 12). The appeal of VADs for precision medicine is enhanced by the potential for new discoveries using forward and reverse genetics, and recognition of genetic variability among the various strains within species, which demands tailored treatment.

Critical challenges hampering the clinical use of VAD must be acknowledged. First, an empirical or “trial-and-error” approach to VAD prescribing will be extremely wasteful given their high cost and may delay the use of more appropriate treatment. Second, conventional drug susceptibility testing approaches are not useful for VAD selection. We consider how these challenges may be addressed and outline new opportunities in the application of pathogen genomics in facilitating the rational use of advanced therapeutics for infectious diseases.

## Advanced Therapeutics Targeting Bacterial Virulence

### Toxin-Binding Monoclonal Antibodies

Many pathogens excrete potent exotoxins as a major virulence factor (VF) that adversely affects host cells. Recent advances in bioengineering facilitate the design and synthesis of humanized monoclonal antibodies (hMAb) that can specifically target and neutralize these proteins (7, 8, 13). Several therapeutic agents have demonstrated benefit in clinical trials for the treatment of diseases caused by toxin-producing bacteria; for example, Bezlotoxumab (Zinplava^®^) and Actoxumab bind *Clostridium difficile* toxins B and A, respectively. Bezlotoxumab have been used to treat recurrent colitis caused by toxin B producing organisms (13). Urtoxazumab, a hMAb against Shiga-like toxin 2 aims to reduce the risk of hemolytic uremic syndrome in patients infected with Shiga-like toxin-producing *Escherichia coli* (7). Raxibacumab and Obiltoxaximab received accelerated FDA approval for the treatment and prevention of inhalational anthrax, which is a potential bioterrorism agent that may cause rapidly progressive fatal disease (14).

### Microbial Cell-to-Cell Signaling (“Quorum Sensing”) Inhibitors

**Figure 2:**
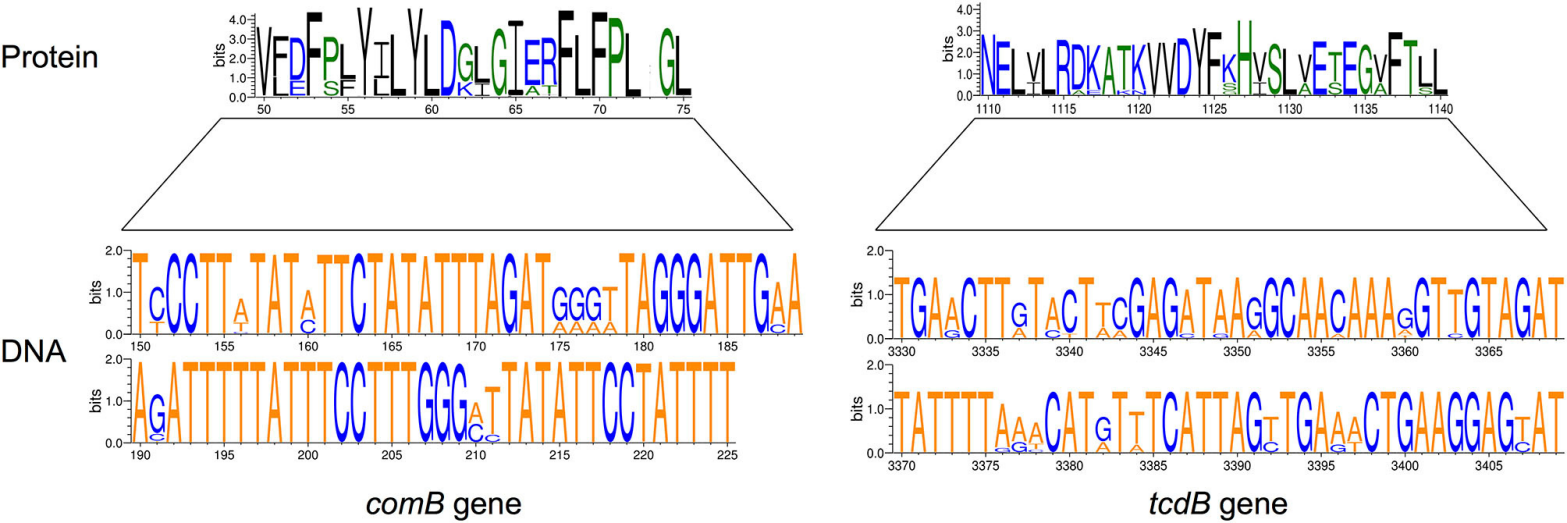
Examples of variation in virulence genes and proteins affecting VAD targets. The size of letters representing nucleotides (DNA) or amino acids (Protein) reflects their relative frequency in individual positions in the VAD target gene. Only 6% of *Streptococcus pneumoniae comB* gene (75-bp of 1,326-bp gene) and 2% of *Clostridium difficile tcdB* gene (90-bp of 7,101-bp gene) are shown; multiple sequence variations with amino acid relevance are present. Amino acids are colored according to their chemical properties: polar amino acids are green, basic blue, and hydrophobic are black. The height of the y-axis indicates the maximum entropy for the given sequence type. Prepared using Weblogo (www.weblogo.berkeley.edu).

Disruption of the collective behavior of bacteria coordinated by small signaling molecules, known as “quorum sensing” (QS, also called density sensing), has been proposed as another anti-virulence strategy (Figure 2). These signaling molecules, which include self-inducing oligopeptides, can activate cellular processes that affect virulence, motility, biofilm formation and drug resistance mechanisms, when produced in sufficient numbers by high-density bacterial populations. Quorum sensing inhibition or quorum quenching (QQ) aims to reduce or destroy the QS signal (15). QS circuits represent hierarchical cross-regulated networks e.g., the *las*-QS circuit positively regulates the *rhl-* and *pqs*-QS circuits. In *Pseudomonas aeruginosa* the QS system consists of two N-acyl-homoserine-lactone (AHL) regulatory circuits (genes *las* and *rhl*) and a non-AHL-mediated QS signaling pathway using hierarchically connected alkyl-4-quinolones (16).

Inhibition of QS can be achieved by interference with the QS signal biosynthesis, inactivation of the QS signals or blocking of QS signal detection by receiving receptors. Inactivation of QS signals after they have been secreted can be achieved either by QQ enzymes, monoclonal antibodies or synthetic polymers modifying or binding to autoinducer proteins. A number of small molecules have demonstrated the ability to interfere with QS networks (16, 17). QS signal detection inhibitors, such as Furanone C-30, are structural analogs that bind to the receptor. Antagonists of particular QS signals affect the signaling cascade and exert inhibitory activity on the whole QS network (17). Numerous QQ agents have been identified that inhibit key QS pathways, AHL synthesis or AHL receptors in *P. aeruginosa* and other bacteria. However, the majority of QQ drugs remain in pre-clinical stages with some repurposed drugs in clinical trials (Figure 1) (18).

### Adhesion and Injectisome Blockers

The interruption of bacterial adhesion to host cells appeals as a strategy to slow cellular invasion and buy time for the immune system to mount an effective response (19, 20). Attachment proteins on the surface of bacteria are critical to bind to cell receptors and are primary targets for the development of therapeutic antibodies, monobodies (small target binding proteins), or small molecules that delay or prevent bacterial adhesion. Monoclonal antibodies against fimbrial adhesin FimH of uropathogenic *Escherichia coli* can displace the ligand from the binding pocket and thereby block bacterial adhesion necessary for surface-adherent biofilms, facilitating treatment of recurrent urinary tract infections (21). Small molecules with the ability to inhibit adhesion and effector proteins secreted by bacterial secretion systems (SS) have shown promise in animal models (20, 22). For instance, Licoflovanol exhibited strong inhibitory effects on the secretion of Salmonella pathogenicity island 1 (SPI-1)-associated effector proteins via regulating the transcription of the *sicA/invF* genes, without affecting the growth of the bacteria (23).

### Repurposed Drugs With Antivirulence Activity

Potent antivirulence properties have been discovered in medicines that were approved for the treatment of conditions other than bacterial infections. These drugs have established safety and well-understood bioavailability profiles, presenting an appealing treatment option. For example, in *Staphylococcus aureus*, the production of virulence factors is controlled by multiple regulators including SarA, Agr, ArlRS, and the SaeRS two-component system (TCS). The TCS controls the production of over 20 virulence factors including toxins (alpha-hemolysin, gamma-haemolysin, and leukocidins), coagulases, adhesins and different enzymes (e.g., nucleases and proteases). The FDA-approved anti-cancer drugs streptozotocin and floxuridine reduces the virulence of *S. aureus* in experimental models by inhibiting TCS (11).

Mepacrine, a 400-Da acridine derivative, has been used to prevent and treat protozoal infections. Recent recognition of mepacrine's inhibition of enterotoxin produced by *Clostridium perfringens* motivated its inclusion in the list of potential antivirulence drugs (24). Azathioprine, an immunosuppressive drug, also affect 5-aminoimidazole-4-carboxamide ribotide (AICAR) transformylase, an enzyme involved in purine biosynthesis in *P. aeruginosa* and other bacteria. Interestingly, this activity is abolished in an *E*. *coli purH* mutant strain, unable to produce AICAR transformylase (25), but its potential clinical use has not been explored. *In silico* molecular docking demonstrated that the antifungal medications clotrimazole and miconazole, as well as an antibacterial compound active against Gram-positive pathogens, clofoctol, can act as QS inhibitors by targeting the transcriptional regulator PqsR. The most active inhibitor, clofoctol, specifically inhibited the expression of *pqs*-controlled virulence traits in *P. aeruginosa*, such as pyocyanin production, swarming motility, biofilm formation, and expression of genes involved in siderophore production (26). The hypertension drug guanfacine can also act as a potent QS inhibitor for *P. aeruginosa* in biofilms (27), although the mechanism of action requires further elucidation. Antimicrobial effects can be achieved by lower doses of these repurposed drugs than those required for anti-cancer activity or immunosuppression, however, their safety and tolerability profiles as VAD candidates require further elucidation. For example, it might be inappropriate and unsafe to use hypertension or immunosuppression drugs as VAD, even in lower doses, especially in patients with low blood pressure or pre-existing immunosuppression.

### *In silico* Recognition of VADs Targets

The concept of a “druggable genome” was inspired by the identification of proteins as potential targets for novel agents or repurposed drugs using whole genome association studies (28). This concept opens the whole genome to interrogation aimed at predicting susceptibility to all available treatment options (29). The growing uptake of bench-top sequencing instruments by microbiology laboratories greatly improves access to pathogen genomics. The sequencing of clinically relevant bacteria has become appealing from both high-resolution diagnostics and laboratory automation points of view. Whole genome sequencing of bacteria from clinical cultures allows reliable drug susceptibility inference for a range of pathogens, especially those with well-defined resistance conferring mutations such as *Mycobacterium tuberculosis*. Several multi-center studies demonstrated high correlation between phenotypic and genotypic resistance in *M. tuberculosis* (30, 31). The growing size and improved curation of bacterial genome databases strengthen sequence similarity-based detection of VF and enable machine learning classifiers that utilize both genome composition and sequence homology (32, 33). Furthermore, the maturation of bioinformatics tools for bacterial genome assembly and annotation makes the analysis of core and variable genomes and the identification of important virulence markers more feasible (34).

## Importance of VAD for Priority AMR Pathogens

The emergence of bacterial pathogens with multi-drug resistance strengthens the case for exploring alternative therapeutic approaches. Antibiotics are typically prescribed on an empirical basis because of their broad-spectrum antimicrobial activity and the limitations of culture guided clinical decision-making. The resultant misuse of antibiotics has contributed to the current antimicrobial resistance (AMR) crisis. Key examples of high priority AMR pathogens with potential VAD targets and candidate drugs that have demonstrated activity against these AMR pathogens are summarized in Table 1. High-resolution diagnostics are essential for rational VAD selection and current laboratory methods are insufficient to guide optimal therapy choice. The best patient group for using VAD as adjunct therapy to existing antibiotic treatment may be patients with chronic persistent infections caused by antibiotic resistant bacteria. Close alignment of VAD use with existing antibiotic stewardship practices and governance should minimize the risk of misuse threats from acquired resistance.

**Table 1:** VAD candidates for high-burden AMR pathogens.

		**Examples of relevant VAD**			
**Bacterial pathogens**	**AMR status**	**Target**	**VAD**	**VAD mechanisms**	**References**
*Staphylococcus aureus*	ESKAPE pathogen, resistance reported for aminoglycosides, β-lactams, fluoroquinolones, macrolides, and tetracyclines	α-toxin	MEDI4893 Salvecin	Toxin capture and inactivation	(35, 36)
*Klebsiella pneumoniae*	ESKAPE pathogen, resistance reported for aminoglycosides, carbapenems, 3G cephalosporins, fluoroquinolones, Polymyxins, tetracyclines	MrkA	Antibody KP3	Binds to MrkA, reduced binding to epithelial cells and biofilm formation	(37)
*Acinetobacter baumannii*	ESKAPE pathogen, resistance reported for β-lactams, carbapenems, cephalosporins, polymyxins, tigecycline	Type 4 Pilli	Virstatin	Decrease pilliation and perturbs adhesion and prevent biofilm	(38)
*Pseudomonas aeruginosa*	ESKAPE pathogen, resistance reported for aminoglycosides, cephalosporins, carbapenems, piperacillin-tazobactam, polymyxins, fluoroquinolones	Quorum Sensing	5-fluorouracil	Biofilm repression and attenuation of quorum sensing phenotypes	(39)
Uropathogenic *E. coli*	Globally disseminated ST131 reported to be resistant to fluoroquinolones and extended spectrum cephalosporins	TonB iron uptake system	Compound 120304	Binds to TonB, blocking iron uptake	(10)
*Mycobacterium tuberculosis*	Multidrug resistance associated with resistance to isoniazid and rifampicin	Heparin binding Haemagglutinin	4,057	Binds to HBHA and blocks adhesion	(40)
*Vibrio vulnificus*	Opportunistic pathogen. Reports of antibiotic resistance reported globally	HlyU, transcriptional regulator	Fursultiamine hydrochloride	Inhibits HlyU regulated toxin genes	(12)
*Vibrio cholerae*	Multi and extensive drug resistance reported.	Quorum sensing	Compound 18	Agonist of quorum sensing which would lead to reduced virulence	(41)
*Salmonella enterica*	Multidrug resistance reported for multiple serovars, including Salmonella Typhi	SPI-1 Type 3 Secretion System effectors	Licoflavonol	Inhibits secretion of SPI-1 effector proteins by reducing expression of *sicA* and *invF*	(23)
*Streptococcus pneumoniae*	Resistance reported for β-lactams, chloramphenicol, fluoroquinolones, macrolides, and tetracycline	Toxins	CAL02	Acts as a decoy to sequester toxins	(42)
*Clostridium difficile*	Proliferation due to microbial overgrowth from exposure to antibiotics	Toxin B	Bezlotoxumab	Toxin capture and inactivation	(13)
Shiga Toxin producing *E. coli*	Reported strains with extended spectrum beta-lactamases	Shiga toxin	Urtoxazumab, Shigamab	Toxin capture and inactivation	(7, 43)

## Pathogen Genomics Guided Precision Medicine

The precision of VAD selection within species of bacterial pathogens can be achieved by using comparative and subtractive genomics approaches. This *in silico* can identify species- and strain-specific genes or groups of genes that are responsible for a unique phenotype (i.e., virulence) and can be targeted by different classes of VAD. It is widely accepted that the virulence of bacterial pathogens can be predicted from their genomes, once the full diversity of VF has been documented (33). Genomic determinants of virulence can be classified into genes responsible for host damage, genes coding for proteins regulating the expression of these VF genes, and so called “life-style” genes enabling colonization, intracellular survival or immune system evasion. Thus, routine sequencing of bacteria responsible for infections of high consequence offers an opportunity to identify the presence or absence of VADs targets and mutations that may modify pathogen susceptibility to supplementary treatment.

While VADs possess no bactericidal activity there is emerging evidence that resistance mutations can occur, since VAD treatment may indirectly affect bacterial survival (44, 45). For example, *in vitro* resistance to QS inhibitor, furanone C-30, arose rapidly in clinical strains of *P. aeruginosa* due to silencing mutations in the *mexR* gene (44). This gene is a negative regulator of efflux pumps and is involved in the export of the C12-HSL autoinducer protein. Furthermore, as a number of significant VF are coded by genes located on plasmids and prophages, gene loss and gene acquisitions occur frequently. Indeed, plasmid flux occurs quickly, both independent of and much faster than the slow accumulation of single nucleotide polymorphisms (SNPs) (46). While not documented thus far, the risks of strain replacement following VAD administration requires consideration and laboratories should test for resistance after repeated VAD exposure.

Effects of mutations or sequence variations in VF coding regions require more nuanced methods of assessment, both computational and phenotypic. Within-host pathogen heterogeneity is problematic, since gene sequence diversity among bacterial meta-populations can hide target protein conformational changes in minority populations; as demonstrated for common pathogens *Salmonella typhimurium* and *Streptococcus pneumoniae* (47, 48). Sequencing of different *S. typhimurium* sequence types (ST19, ST34, and ST36) revealed a pool of 152 virulence genes and 79 virulence signatures (49), and examination of VAD targets suggest highly variable efficacy in different sequence types. Within species variability of VAD targets includes the absence of coding genes and/or variation in their sequences. For example, within species identity of *lasI/lasR* genes in *Pseudomonas aeruginosa* and *comD* gene in *Streptococcus pneumoniae* targeted by QS inhibitors can be, in some clinical strains, as low as 94.5 and 57.8%, respectively (Figure 2).

**Figure 3:**
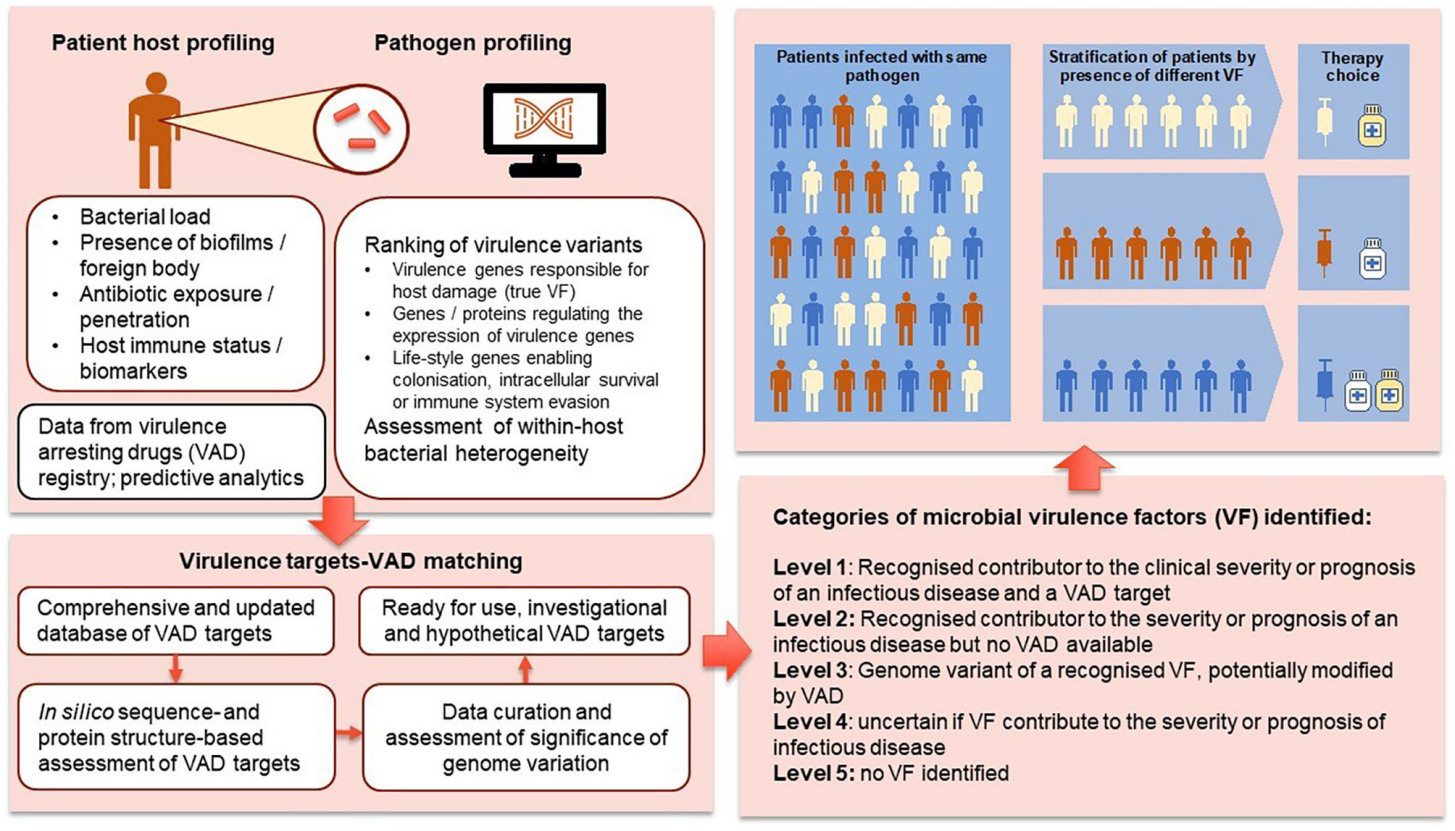
Proposed pathway for using genomic profiling for VAD selection.

Sequencing of cancers has become a mainstay in oncology with a proliferation of advanced therapeutics targeting tumor-specific gene mutations. Personalized cancer care is enabled by the development of data analytics to assess the treatment implications associated with specific mutants and linking patients to optimal therapeutic approaches (29, 50–52). We are now in a position to extend the precision medicine paradigm to infectious diseases. Figure 3 outlines the proposed pathway for using genomic profiling for intelligent VAD selection and precision infectious diseases (ID) medicine. We suggest four levels of significance for VF-associated genome structures: level 1—recognized contributor to the clinical severity or prognosis of an infectious disease and a VAD target; level 2—recognized contributor to the severity or prognosis of an infectious disease but no VAD available; level 3—genome variant of a recognized VF potentially modified by VAD; level 4—uncertain if VF contribute to the severity or prognosis of infectious disease.

## Genomics Guided VAD Utilization Models

The narrow spectrum of targeted VAD, the tolerability profiles of repurposed VADs, as well as their high cost, indicate that contemporary antimicrobial utilization models are inadequate. We need to move toward a highly-tailored approach to ensure clinical- and cost-effective use of these novel therapeutics. The standard approach using minimum inhibitory concentrations (MIC) as predictors of clinical response to antibiotics cannot be extrapolated to VAD use and MIC-based PK/PD targets are not relevant. Novel approaches are needed to match specific virulence properties of an individual pathogen to the optimal mix of treatment modalities available. The lack of methods to enable the most effective (including cost-effective) use of such tailored treatment limits clinical uptake. We argue that inferences about VAD susceptibility requires microbial genomics. Table 2 illustrates a hierarchy of genomic variants that can be relevant for such inferences, using automated high-quality genome assembly and annotation to accurately reconstruct the accessory genome (46). However, the presence/absence or expression variability of VAD targets may be insufficient for accurate *in silico* determination of susceptibility (44), and a synthesis of genomic, transcriptomic and bacterial phenome data may be required to optimize treatment recommendations. High-resolution genome sequence analysis can also illuminate within-host heterogeneity of bacterial populations. Such an approach would form an important nucleus of knowledge upon which we can assemble a much larger strategy for value-based use of advanced therapeutics. This approach will require bioinformaticians to work closely with pathologists and clinicians to identify the most therapeutic options.

**Table 2:** Example of an actionable hierarchy for identified genomic variants.

**Hierarchical level^a^**	**Presence of VAD target**	**Examples of potential clinical applications^b^**		
		**Genome analysis**	**Genome category**	**Interpretation**
A pathogen with corresponding FDA-approved VAD agents	Variant lacking the VAD target	Absence of *toxB* gene or its promoter in *C. difficile* recovered from a patient with recurrent/severe colitis	Level 5	Bezlotoxumab (13) use not indicated
	Variant with the VAD target	Presence of exotoxin genes *pagA, lef* and *cya* on virulence plasmid pXO1 in *Bacillus anthracis* from a patient with suspected inhalational anthrax	Level 1	Raxibacumab (14) use indicated
A pathogen with corresponding FDA-approved repurposed agents	Variant lacking the VAD target	Mutation in *mexR* transcriptional repressor of *P. aeruginosa* from a patient with a recurrent urinary tract infection	Level 3	Furanone C-30 (16, 17) not to be considered
	Variant with the drug target	Wild-type T3SS in *Salmonella* recovered from a patient with chronic carriage	Level 1	Licoflovanol (23) to be considered
A pathogen with corresponding agent in a clinical trial	Variant lacking the VAD target	A silencing mutation in *stx*_2_ gene of *Escherichia coli* from a patient with Shiga-toxin positive diarrhea	Level 3	Shigamab (43) not to be considered
	Variant with the drug target	Wild-type *hly* gene and its promoter in *S. aureus* from a patient with pneumonia/empyema	Level 2	Salvecin (36) to be considered

a *Hierarchy of actionable genomic variants, ranging from cases with the strongest evidence base supporting cause and effect to the weakest*.

b *The examples provided translate predicted resistance or sensitivity to a clinical recommendation*.

Microbial genomics can stratify a pathogen's likely VAD response by comparing the sequenced genome to a reference genome with known susceptibility or resistance to different classes of VAD. It is important to ensure availability of these reference genomes to diagnostic laboratories for timely *in silico* selection of the most appropriate VAD. While several pipelines for genomics surveillance and drug resistance determination have been implemented in practice (30, 52, 53), genomic inferences focused on VAD susceptibility are underdeveloped (38). It does not come as a surprise that, given the high complexity and cost of advanced therapeutics, the establishment of appropriate national registries has been a condition of their approval by the FDA/EMA in other domains. Effective post-registration monitoring and the establishment of a VAD register is important to record the indications and outcomes of VAD administration, as well as relevant pathogen details. This register will integrate available data, including relevant host data to develop predictive analytics to guide VAD use.

“Similarity assessments” may identify patients who experienced a desirable health outcome following VAD treatment and displayed similar clinical characteristics (54). The establishment and curation of databases linking genotypes and phenotypes is essential to improve the consistency of variant interpretation and relevant data sharing may be considered the ethical and legal duty of microbiology laboratories (55, 56). Ideally pathogen genomic data should be integrated into laboratory information systems and electronic health records (EHR). EHR systems have not yet anticipated the complexity and variety of microbial genomic information that needs to be captured, interpreted and acted upon. As with human genomics, improved integration of pathogen genomics data with EHR systems should assist clinical decision making and secondary use of data for translational research. The absence of well-established nomenclatures presents another challenge for integration and standardization as terminology standards developed for human genomics have not been applied with vigor in the infectious disease domain (57, 58).

## Conclusion

The accurate diagnosis of pathogens based on next generation sequencing have substantial potential to improve value-based precision ID medicine. The maturation of genome-scale analysis offers a new prism through which to explore individually targeted therapy. Such an approach will identify the most beneficial treatment for each pathogen based on its genomic profile. Emerging markets for advanced therapeutics are pushing the boundaries of health systems and service delivery models. Better methods to identify patients who are most likely to benefit from expensive novel antimicrobial agents such as VAD, should facilitate regulatory approval and improve clinical uptake. Such a new paradigm is essential for future therapy design, which may also include engineered microbes designed to treat immunological or metabolic disorders, or to inhibit specific pathogens, often with site-specific delivery. The development of *in silico* assessment models is timely as it coincides with the crucial early stages of VAD roll out, where optimal systems are required to optimize its clinical value and cost effectiveness. It will augment antimicrobial stewardship and pharmacovigilance for advanced therapeutics, provide crucial policy guidance to regulating agencies, assist hospitals to optimize the use of these expensive medicines and create market opportunities for biotech companies and microbiology laboratories.

## Data Availability Statement

The original contributions presented in the study are included in the article/supplementary material, further inquiries can be directed to the corresponding author/s.

## Author Contributions

VS and BM designed the manuscript. All co-authors contributed to writing the manuscript and production of figures.

## Conflict of Interest

The authors declare that the research was conducted in the absence of any commercial or financial relationships that could be construed as a potential conflict of interest.
